# Neuroprotective Effect of Apolipoprotein D in Cuprizone-Induced Cell Line Models: A Potential Therapeutic Approach for Multiple Sclerosis and Demyelinating Diseases

**DOI:** 10.3390/ijms22031260

**Published:** 2021-01-27

**Authors:** Eva Martínez-Pinilla, Núria Rubio-Sardón, Rafael Peláez, Enrique García-Álvarez, Eva del Valle, Jorge Tolivia, Ignacio M. Larráyoz, Ana Navarro

**Affiliations:** 1 Department of Morphology and Cell Biology, University of Oviedo, 33003 Oviedo, Spain; nuria199510@hotmail.com (N.R.-S.); garal.enrique@gmail.com (E.G.-Á.); valleeva@uniovi.es (E.d.V.); anavarro@uniovi.es (A.N.); 2 Instituto de Neurociencias del Principado de Asturias (INEUROPA), 33003 Oviedo, Spain; 3 Instituto de Investigación Sanitaria del Principado de Asturias (ISPA), 33011 Oviedo, Spain; 4 Biomarkers and Molecular Signaling Group, Neurodegeneration Area, Center for Biomedical Research of La Rioja (CIBIR), 26006 Logroño, Spain; rpelaez@riojasalud.es (R.P.); ilarrayoz@riojasalud.es (I.M.L.)

**Keywords:** breast cystic fluid, clozapine, endocytosis, glia, neurons, ROS

## Abstract

Apolipoprotein D (Apo D) overexpression is a general finding across neurodegenerative conditions so the role of this apolipoprotein in various neuropathologies such as multiple sclerosis (MS) has aroused a great interest in last years. However, its mode of action, as a promising compound for the development of neuroprotective drugs, is unknown. The aim of this work was to address the potential of Apo D to prevent the action of cuprizone (CPZ), a toxin widely used for developing MS models, in oligodendroglial and neuroblastoma cell lines. On one hand, immunocytochemical quantifications and gene expression measures showed that CPZ compromised neural mitochondrial metabolism but did not induce the expression of Apo D, except in extremely high doses in neurons. On the other hand, assays of neuroprotection demonstrated that antipsychotic drug, clozapine, induced an increase in Apo D synthesis only in the presence of CPZ, at the same time that prevented the loss of viability caused by the toxin. The effect of the exogenous addition of human Apo D, once internalized, was also able to directly revert the loss of cell viability caused by treatment with CPZ by a reactive oxygen species (ROS)-independent mechanism of action. Taken together, our results suggest that increasing Apo D levels, in an endo- or exogenous way, moderately prevents the neurotoxic effect of CPZ in a cell model that seems to replicate some features of MS which would open new avenues in the development of interventions to afford MS-related neuroprotection.

## 1. Introduction

Apolipoprotein D (Apo D) is a well-known lipocalin family member that plays a key role in the transport, metabolism and homeostasis of some lipids due to its ability to bind cholesterol, arachidonic acid, steroids, retinoic acid or anandamide, among other small hydrophobic ligands [1,2,3]. In the past decades, increasing evidence at biochemical and functional level suggested that Apo D acts as an antioxidant, being part of the body’s defense system against oxidative stress, and also as an endogenous neuroprotective agent. Indeed, crystallographic analysis revealed that this 29 kDa glycoprotein comprises an eight-stranded antiparallel β-barrel flanked by a singled α-helix that encloses a fat specific ligand-binding pocket. Moreover, Apo D shows various exposed hydrophobic residues located in three of its extended loops which may contribute to Apo D association with lipids and seem to explain its potential as multiligand and multifunctional protein [4,5]. Most importantly, they have been linked to the ability of Apo D to bind and reduce oxidized lipids, and thereby inhibit radical-propagation of lipid hydroperoxides [6,7,8].

Studies in cell systems reported that different stress signal pathways may modulate Apo D transcription. In fact, stressful stimuli such as H_2_O_2_, Rose Bengal, kainic acid, ultraviolet (UV) light, paraquat or lipopolysaccharide, leading to extended growth arrest and apoptosis, increase Apo D expression in a dose and time-dependent manner [9,10]. The potential neuroprotective and prosurvival roles for Apo D have also been proven in animal models where the overexpression of this protein confers greater protection against oxidative stress and contributes significantly to the regulation of longevity; the experimental lack of Apo D causes opposite results [11,12].

In humans, Apo D is expressed in neural and peripheral tissues, detected in cerebrospinal fluid (CSF), in plasma as an important component of high-density plasma lipoproteins (HDL), and in breast cyst fluid (BCF) [1,2,13,14,15]. In nonpathological conditions of the central and peripheral nervous system (CNS and PNS, respectively), Apo D is widely expressed in neurons, glia (astrocytes, oligodendrocytes (OLGs), and Schwann cells), perivascular cells and pericytes [13,16,17,18], contributing to maintain neuronal homeostasis and myelin extracellular leaflet compaction [19,20]. Remarkably, Apo D is upregulated in neural cells and CSF during aging, and in brains affected by neurodegenerative diseases characterized by cellular stress and excitotoxicity such as multiple sclerosis (MS), Spongiform encephalopathy, Parkinson’s disease (PD), Niemann–Pick disease, or Alzheimer’s disease (AD) as well as psychiatric disorders (schizophrenia and bipolar disorder) [8,21,22].

MS is a devastating neurodegenerative disease that affects more than 2 million young adults worldwide, mainly women, with a complex and unknown etiology [23]. This demyelinating, autoimmune and inflammatory disease is manifested clinically in the form of multiple fully or partially reversible symptomatic episodes (reviewed in [24,25,26]), which reflect the progressive focal degeneration of OLGs and myelin membranes around axons in both white and grey matter areas throughout the brain and spinal cord [27,28,29,30]. Classically, OLG dysfunction has been linked to an exacerbated adaptive immune response, involving the recruitment of autoreactive T cells through a defective and permeable blood–brain barrier (BBB), and the activation of B cells [31,32,33]. The consequent inflammatory process activates microglia, astrocytes, and infiltrated macrophages that are able, in turn, to generate oxidative stress-related molecules as reactive oxygen species (ROS) and reactive nitrogen species (RNS) [34], which promote demyelination, compromise the neuro-axonal functional unit and contribute to the progressive tissue damage in MS [26,35,36]. However and contrary to what was thought, recent evidence shows that the biochemical alteration of myelin could be the initial event that triggers a secondary autoimmune response that results in the demyelinating inflammatory reaction taking place in the diseased brains, the so-called “inside-out” model of MS pathogenesis [37,38,39]. In the last two decades, extensive research has been carried out to find efficacious neuroprotective therapies in an attempt to alleviate symptoms and/or slow down or delay the progression of the MS [26,40,41,42,43,44]. Therefore, it is essential to know the root cause of the MS pathology in order to properly select the target for developing efficacious therapeutic interventions. For this purpose, a number of neurotoxin-induced in vivo and in vitro models of demyelination and MS-related neurodegeneration are used. Among all, neuronal and glial cell lines exposed to cuprizone (CPZ), a copper chelator that reversibly impacts on mitochondrial function, may be a convenient experimental approach instrumental in the advance of understanding of the functioning of the nervous system [45,46,47,48].

Previous studies showed that Apo D is upregulated in the CSF of MS patients [49,50], reactive astrocytes, and exhibits a characteristic expression pattern in MS lesions of the brain [51]. In this regard, levels of OLG-derived Apo D are lower in demyelinating plaques but appear to recover in areas of remyelination [51]. This study aims to assess the potential of Apo D (either by triggering its endogenous synthesis or by its exogenous addition), as well as its mechanism of action, to prevent the neurotoxic effect of CPZ in two cell models that mimic biochemical features of MS.

## 2. Results

### 2.1. Apo D Expression in HOG and SH-SY5Y Cells in Response to Cuprizone Treatment

**Figure 1:**
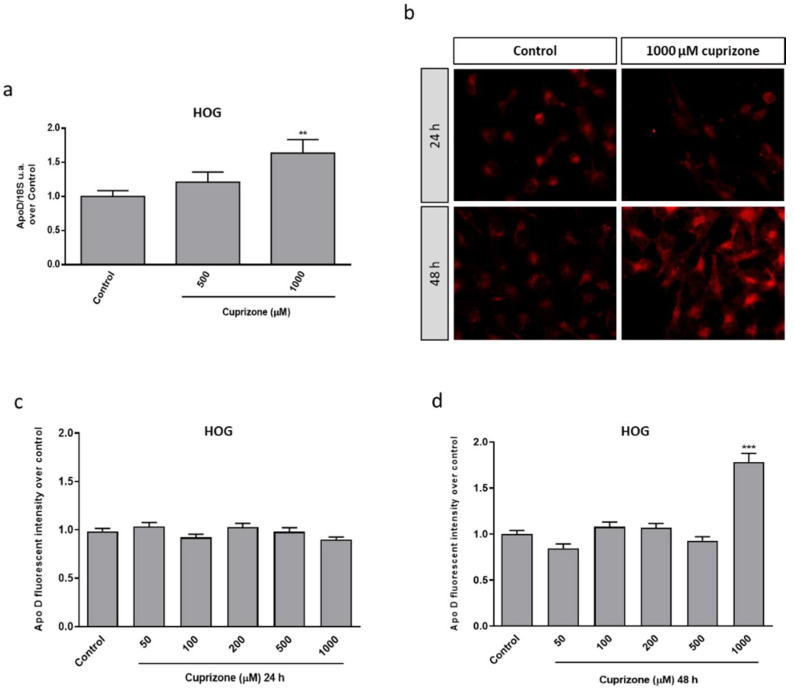
Relative Apo D gene expression in HOG cells treated with 0–1000 μM of CPZ following 24 h. Data represent the quotient between the gene and the expression of the housekeeping gene 18S rRNA. Bars represent the mean ± SEM of all measurements (*n* = 6–8) (**a**). Representative fluorescence microscopy images of Apo D levels in HOG cells treated or not with 1000 μM of CPZ during 24 and 48 h. 40× magnification (**b**). Densitometric quantification of Apo D immunocytochemical signal after 24 (**c**) and 48 h (**d**) of treatment with increasing concentrations of CPZ (50–1000 μM) in HOG cells (*n* = 6). Bars represent mean density per cell in a 40× field ± SEM (over control). Significant differences were analyzed by a one-way ANOVA followed by post-hoc Tukey’s test. ** *p* < 0.01, *** *p* < 0.001 compared with control.

Taking advantage of our experience in the CPZ-induced cell model, some previous results showing that CPZ was able to induce cytotoxic damage mediated by a mitochondrial dysfunction in the HOG and SH-SY5Y cell lines (data not shown), and the findings reported in this work (see next figures), we aimed to analyze the potential effect of CPZ on Apo D expression in these oligodendroglioma and neuroblastoma cell lines by qRT-PCR and immunocytochemistry. As shown in Figure 1, the analysis of Apo D gene expression (Figure 1a) and the immunosignal quantification (Figure 1b–d) revealed that CPZ induced changes in Apo D expression and, interestingly, only at the highest concentration (1000 µM) and at 48 h of treatment. In fact, a constant and almost invariable fluorescence signal was observed in control and treated cells (Figure 1b).

**Figure 2:**
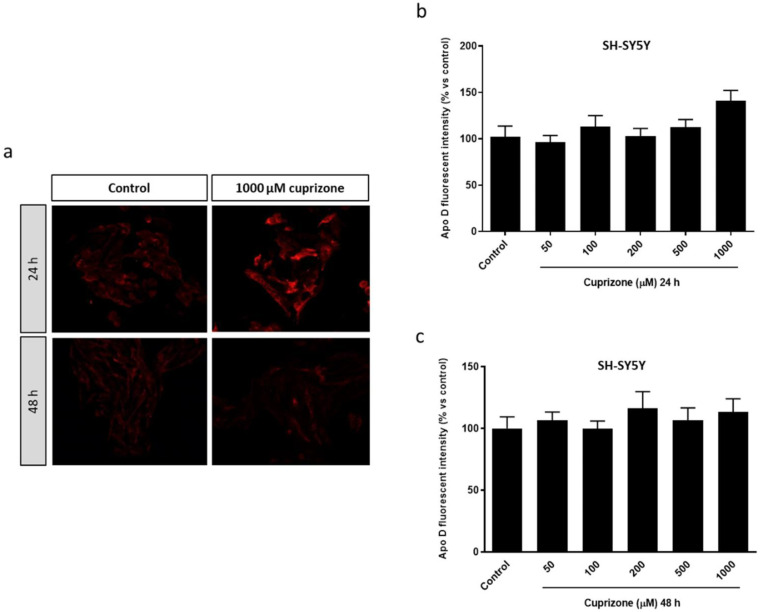
Representative fluorescence microscopy images of Apo D levels in SH-SY5Y cells treated or not with 1000 μM of CPZ during 24 and 48 h. 40× magnification (**a**). Densitometric quantification of Apo D immunocytochemical signal after 24 (**b**) and 48 h (**c**) of treatment with increasing concentrations of CPZ (50–1000 μM) in SH-SY5Y cells (*n* = 6). Bars represent mean density per cell in a 40× field ± SEM (% versus control).

As expected in the case of SH-SY5Y neuroblastoma cells, which according to previous studies show a negligible expression of Apo D [52], we found that these cells exhibited a very scarce endogenous expression of Apo D only detected by immunocytochemistry, and that CPZ did not influence the apolipoprotein synthesis as observed in the images (Figure 2a) and the immunocytochemical quantification (Figure 2b,c).

### 2.2. Clozapine Prevents Loss of Mitochondrial Functionality and Cell Viability in Oligodendroglial and Neuronal CPZ-Induced Models of MS

**Figure 3:**
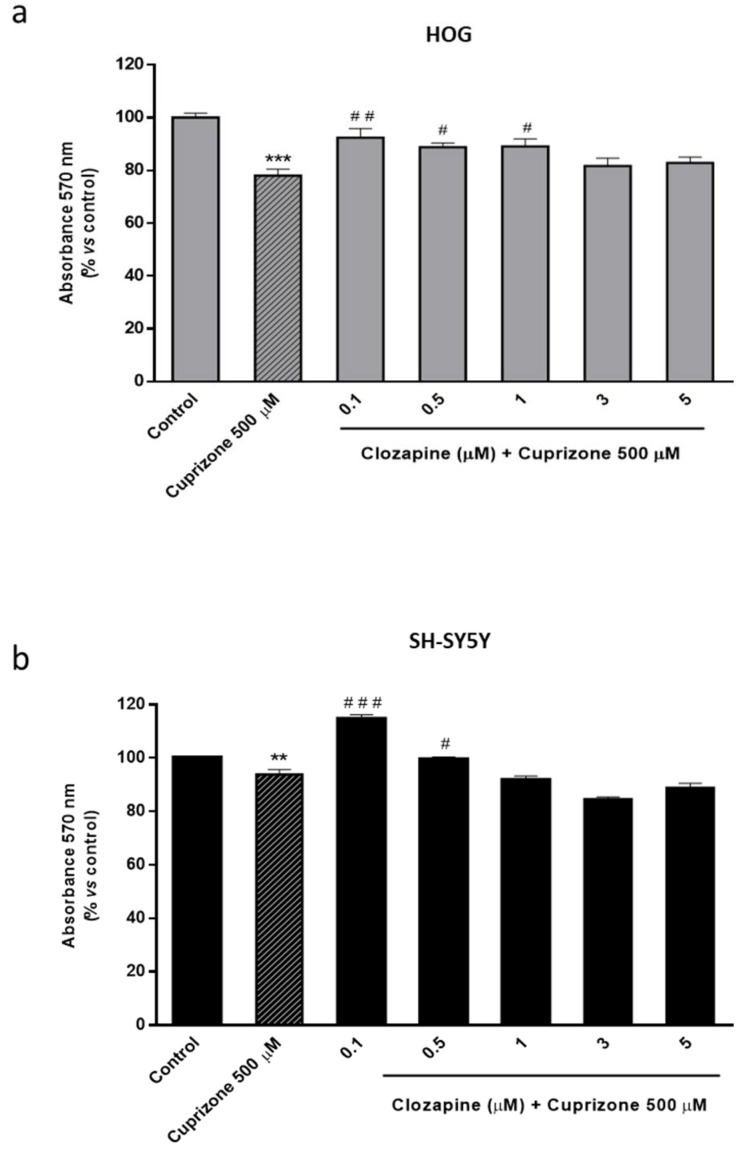
MTT assay in HOG (**a**) and SH-SY5Y cells (**b**) treated with increasing concentrations of CLO (0.1–5 μM) followed by 24 h with 500 µM of CPZ. Cell damage is represented as the percentage of viability versus control. Data are the mean ± SEM of five independent experiments. Significant differences were analyzed by a one-way ANOVA followed by post-hoc Tukey’s test. ** *p* < 0.01, *** *p* < 0.001 compared with control; ^#^ *p* < 0.05, ^##^ *p* < 0.01, ^###^ *p* < 0.001 compared with CPZ treatment.

**Figure A1:**
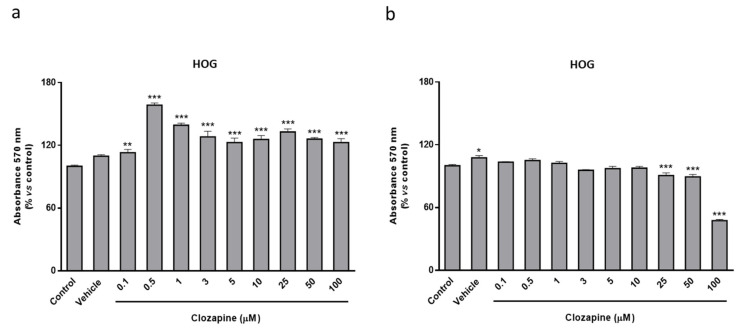
MTT assay in HOG cells treated with increasing concentrations of CLO (0.1–100 μM) during 24 h (**a**) and 48 h (**b**). Cell damage is represented as the percentage of viability versus control. Data are the mean ± SEM of five independent experiments. Significant differences were analyzed by a one-way ANOVA followed by post-hoc Tukey’s test. * *p* < 0.05, ** *p* < 0.01, *** *p* < 0.001 compared with control.

**Figure A2:**
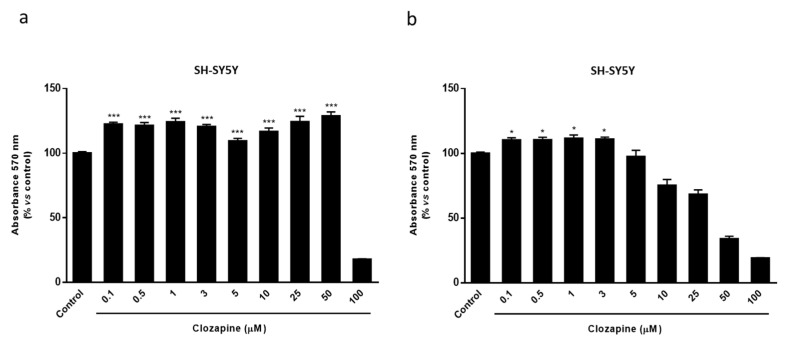
MTT assay in SH-SY5Y cells treated with increasing concentrations of CLO (0.1–100 μM) during 24 h (**a**) and 48 h (**b**). Cell damage is represented as the percentage of viability versus control. Data are the mean ± SEM of five independent experiments. Significant differences were analyzed by a one-way ANOVA followed by post-hoc Tukey’s test. * *p* < 0.05, *** *p* < 0.001 compared with control.

The atypical antipsychotic drug, clozapine (CLO), widely used in the treatment of schizophrenia, among other psychiatric disorders, is considered as a therapeutic agent that seems to exert its beneficial effects by its ability to increase Apo D levels in the brain [53,54]. Therefore, we first evaluated the potential neuroprotective effect of CLO in the CPZ-induced cell models. For this purpose, a wide range of CLO concentrations, from 0.1 to 100 µM, was used to treat HOG or SH-SY5Y cells during 24 and 48 h in absence of CPZ. Once it was established that CLO did not cause loss of cell viability, except in extremely high doses and/or prolonged exposures (Figure A1 and Figure A2), we assessed whether the addition of CLO could avoid the CPZ cytotoxicity. Of note, the two cell lines were differentially affected by CLO, being neurons more sensitive than glial cells to the same concentrations. Our findings demonstrated that CLO was able to prevent the mitochondrial dysfunction caused by the toxic in both HOG and SH-SY5Y cells. As shown in Figure 3, cell viability assessed by the MTT assay revealed that CLO (0.1–1 µM) prevented about 15–30% loss of cell viability when added 24 h before 500 µM of CPZ (Figure 3a,b). Similar results were obtained when cells were treated with CLO and CPZ at the same time. In contrast, this neuroprotective effect was not noticeable when cells were incubated with 500 µM of CPZ for 24 h and subsequently with increasing concentrations of CLO for, at least, another 24 h (data not shown).

### 2.3. Neuroprotective Doses of Clozapine Increase Apo D Expression in the CPZ-Induced Cell Models of MS

**Figure 4:**
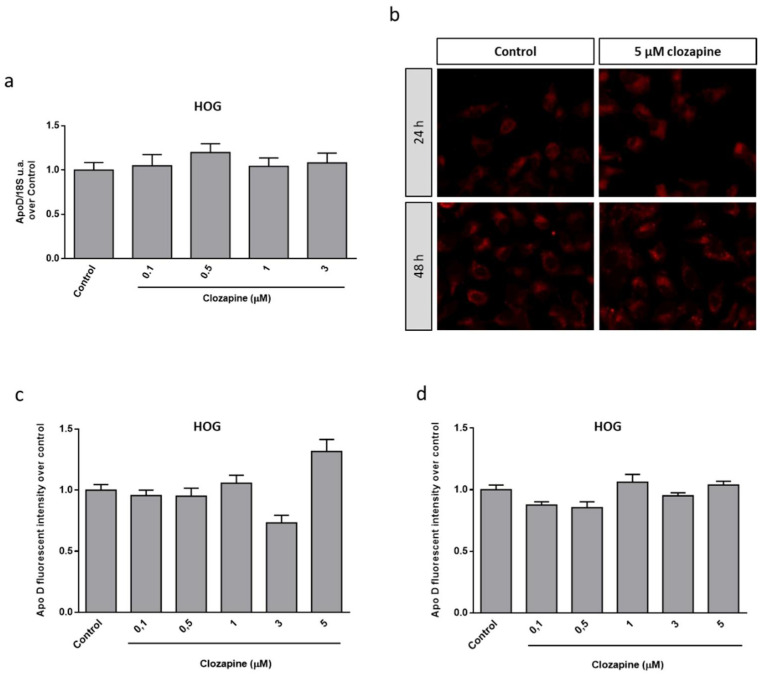
Relative Apo D gene expression in HOG cells treated or not with 5 μM of CLO during 24 h. Data represent the quotient between the gene and the expression of the housekeeping gene 18S rRNA. Bars represent the mean ± SEM of all measurements (*n* = 6–8) (**a**). Representative fluorescence microscopy images of Apo D expression in HOG cells treated or not with 3 μM of CLO during 24 and 48 h. 40× magnification (**b**). Densitometric quantification of Apo D immunocytochemical signal after 24 (**c**) and 48 h (**d**) of treatment with increasing concentrations of CLO (0.1–5 μM) in HOG cells (*n* = 6). Bars represent mean density per cell ± SEM (over control) in a 40× field.

**Figure 5:**
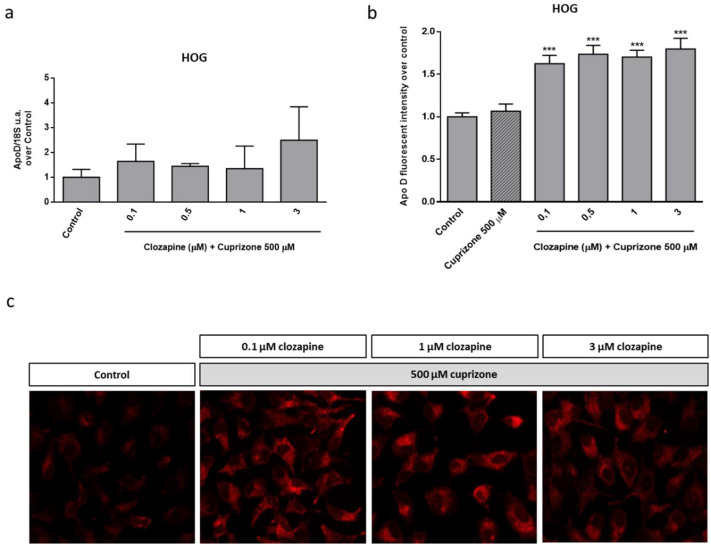
Relative Apo D gene expression in HOG cells treated with increasing concentrations of CLO (0.1–3 μM) during 24 h followed by 24 h with 500 µM of CPZ. Data represent the quotient between the gene and the expression of the housekeeping gene 18S rRNA. Bars represent the mean ± SEM of all measurements (*n* = 6–8) (**a**). Densitometric quantification of Apo D immunocytochemical signal after 24 h of treatment with increasing concentrations of CLO (0.1–3 μM) followed by 24 h with 500 µM of CPZ (*n* = 6). Bars represent mean density per cell in a 40× field ± SEM (over control) (**b**). Representative fluorescence microscopy images of Apo D expression in HOG cells treated with increasing concentrations of CLO (0.1–3 μM) followed by 24 h with 500 µM of CPZ. 40× magnification (**c**). Significant differences were analyzed by a one-way ANOVA followed by post-hoc Tukey’s test. *** *p* < 0.001 compared with control.

Then, and in order to check the possible link between the neuroprotective effect observed for CLO and the endogenous Apo D levels, the expression of this apolipoprotein was analyzed in HOG cells upon CLO treatment. qPCR and immunocytochemical analyses demonstrated that this antipsychotic drug did not produce changes in Apo D expression by itself, at least in the tested concentrations and times of treatment (Figure 4). However, CLO (0.1–3 µM) induced an increase in Apo D synthesis when it was coadministrated with CPZ in OLGs at the same concentrations that prevented the loss of viability caused by the toxin. As shown in Figure 5, the increase in Apo D signal was higher than the control values when added 24 h before 500 µM of CPZ.

**Figure 6:**
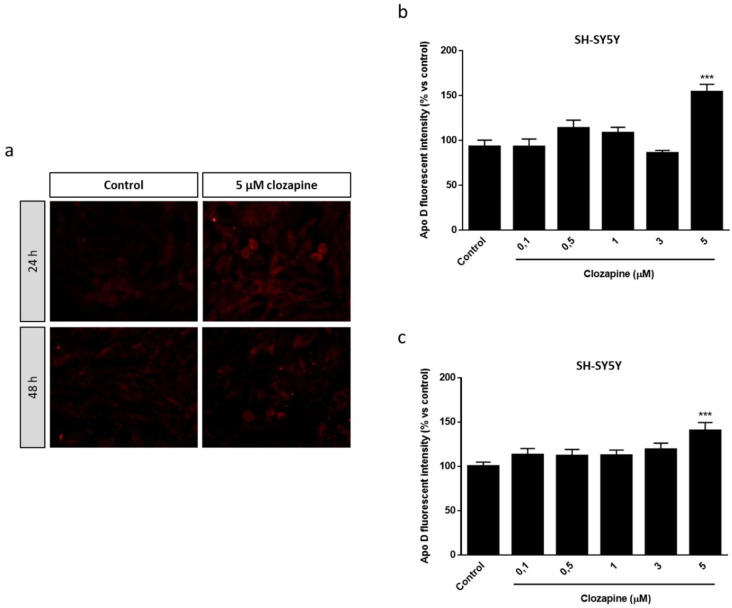
Representative fluorescence microscopy images of Apo D expression in SH-SY5Y cells treated or not with 5 μM of CLO during 24 and 48 h. 40× magnification (**a**). Densitometric quantification of Apo D immunocytochemical signal after 24 (**b**) and 48 h (**c**) of treatment with increasing concentrations of CLO (0.1–5 μM) in SH-SY5Y cells (*n* = 6). Bars represent mean density per cell ± SEM (% versus control) in a 40× field. Significant differences were analyzed by a one-way ANOVA followed by post-hoc Tukey’s test. *** *p* < 0.001 compared with control.

**Figure 7:**
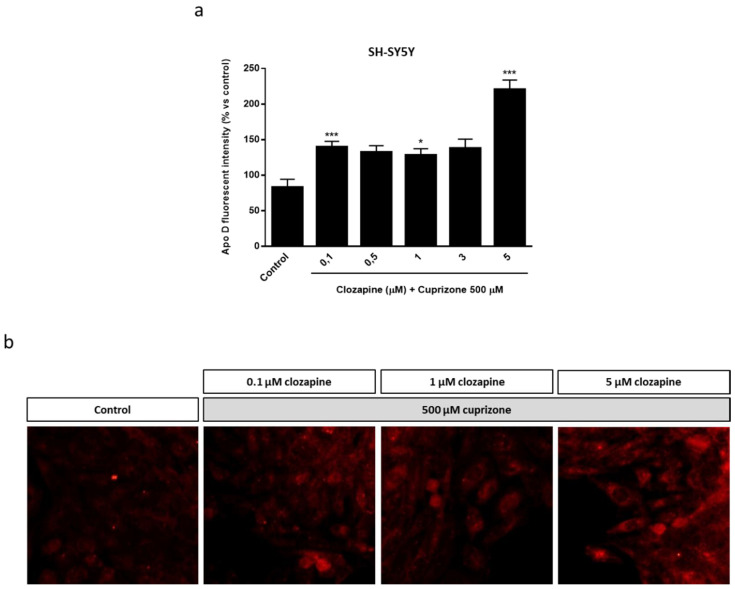
Densitometric quantification of Apo D immunocytochemical signal in SH-SY5Y cells after 24 h of treatment with increasing concentrations of CLO (0.1–5 μM) followed by 24 h with 500 µM of CPZ (*n* = 6). Bars represent mean density per cell in a 40× field ± SEM (% versus control). Significant differences were analyzed by a one-way ANOVA followed by post-hoc Tukey’s test. * *p* < 0.05, *** *p* < 0.001 compared with control (**a**). Representative fluorescence microscopy images of Apo D expression in SH-SY5Y cells treated with increasing concentrations of CLO (0.1–5 μM) followed by 24 h with 500 µM of CPZ. 40× magnification (**b**).

Similar results, but with some nuances, were obtained in the neuroblastoma cell line. In fact, immunocytochemical assays revealed that CLO induced changes in Apo D expression in SH-SY5Y cells but only at the highest concentration (5 µM), at 24 and 48 h of treatment, as observed in the images (Figure 6a) and the immunocytochemical quantification (Figure 6b,c). When CLO was added 24 h before 500 µM of CPZ the Apo D immunosignal increased from 1.5 to 2-fold (compared to control) in the concentrations of the antipsychotic drug associated with the neuroprotective effects (Figure 7a,b). Interestingly, the treatment with 5 µM of CLO, which almost doubled Apo D levels in SH-SY5Y cells (Figure 7), was unable to prevent the cytotoxic effect of CPZ (Figure 3b). 

### 2.4. Neuroprotective Effect of the Exogenously Added hApo D in Oligodendroglial and Neuronal CPZ-Induced Models of MS

**Figure 8:**
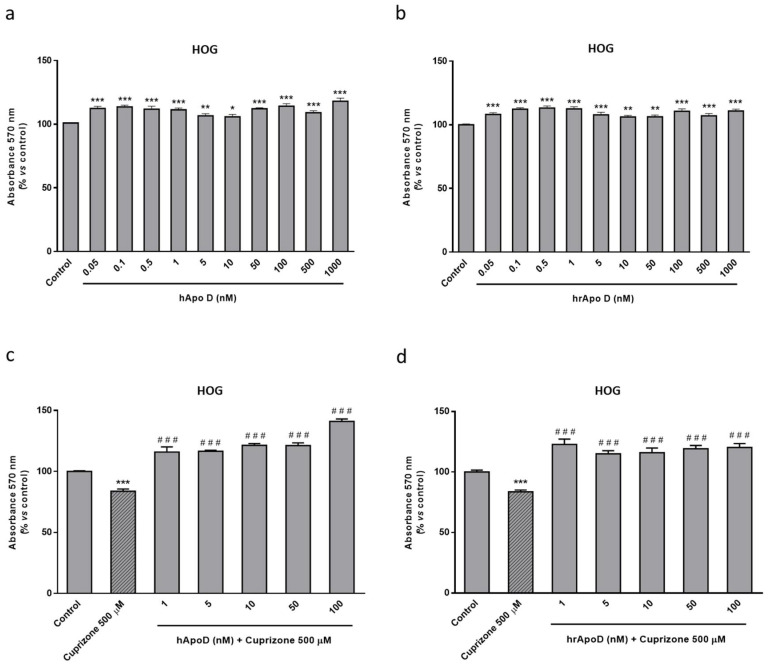
Top panel: MTT assays in HOG cells treated with increasing concentrations (0.05–1000 nM) of hApo D (**a**) or of hrApo D during 24 h (**b**). Bottom panel: MTT assays in HOG cells treated with increasing concentrations (1–100 nM) of hApo D (**c**) or of hrApo D (**d**) for 24 h followed by 24 h with 500 µM of CPZ. Cell damage is represented as the percentage of viability versus control. Data are the mean ± SEM of five independent experiments. Significant differences were analyzed by a one-way ANOVA followed by post-hoc Tukey’s test. * *p* < 0.05, ** *p* < 0.01, *** *p* < 0.001 compared with control; ^###^ *p* < 0.001 compared with CPZ treatment.

**Figure 9:**
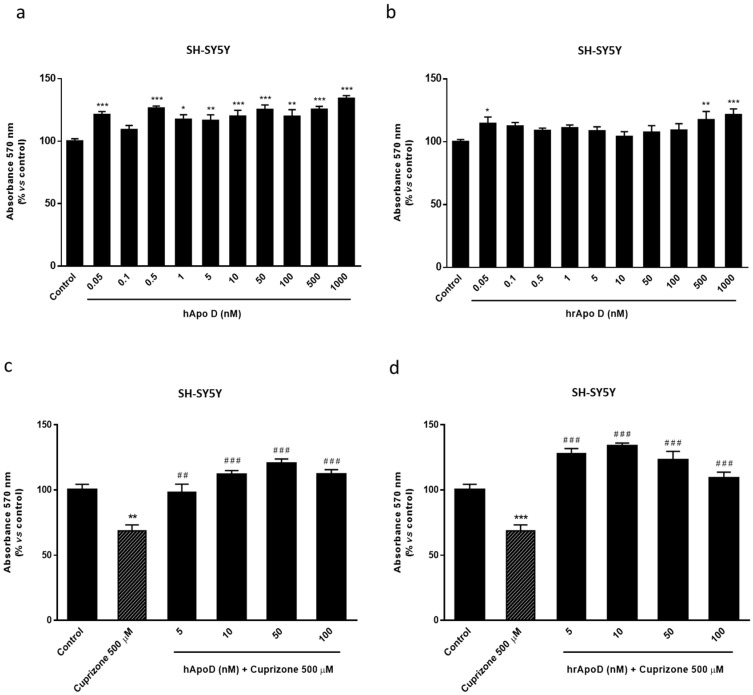
Top panel: MTT assays in SH-SY5Y cells treated with increasing concentrations (0.05–1000 nM) of hApo D (**a**) or of hrApo D (**b**) during 24 h. Bottom panel: MTT assays in SH-SY5Y cells treated with increasing concentrations (1–100 nM) of hApo D (**c**) or of hrApo D (**d**) for 24 h followed by 24 h with 500 µM of CPZ. Cell damage is represented as the percentage of viability versus control. Data are the mean ± SEM of five independent experiments. Significant differences were analyzed by a one-way ANOVA followed by post-hoc Tukey’s test. * *p* < 0.05, ** *p* < 0.01, *** *p* < 0.001 compared with control; ^##^ *p* < 0.01, ^###^ *p* < 0.001 compared with CPZ treatment.

The next step to assess the neuroprotective potential of Apo D was to check the impact of the exogenous addition of human Apo D (hApo D), hApo D purified from BCF or human recombinant Apo D (hrApo D), in the CPZ-based cell models of MS. On the one hand, we found that both apolipoproteins induced some improvement in mitochondrial oxidation and, consequently, an increase in OLGs (Figure 8) and neurons (Figure 9) viability under normal conditions. On the other hand, the analysis revealed that treatment with hApo D (5–100 nM) totally prevented the loss of viability caused by the addition of 500 µM CPZ for 24 h in the HOG cells (Figure 8c). Although to a lesser extent, similar results were found in cells pretreated with hrApo D (Figure 8d). Noteworthy, these findings were confirmed in the SH-SY5Y neuroblastoma cells that lack endogenous Apo D expression. Accordingly, both hApo D and hrApo D were able to prevent the toxic effect of CPZ after 24 h of treatment in neuroblastoma cells as well (Figure 9c,d).

**Figure 10:**
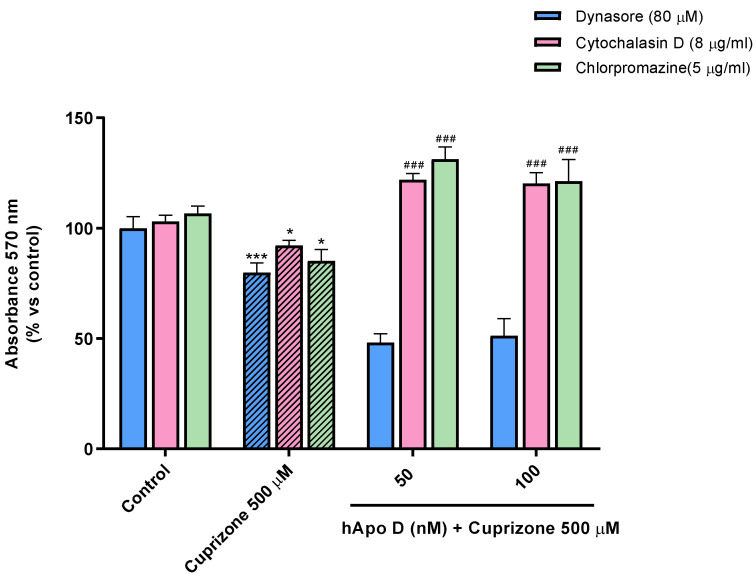
MTT assay in SH-SY5Y cells treated with 8 µg/mL cytochalasin D, 5 µg/mL chlorpromazine hydrochloride, or 80 µM dynasore prior to the addition of increasing concentrations (50–100 nM) of hApo D for 24 h followed by 24 h with 500 µM of CPZ. Cell damage is represented as the percentage of viability versus control. Data are the mean ± SEM of five independent experiments. Significant differences were analyzed by a one-way ANOVA followed by post-hoc Tukey’s test. * *p* < 0.05, *** *p* < 0.001 compared with control; ^###^ *p* < 0.001 compared with CPZ treatment.

Finally, and in order to test whether Apo D exerts its antioxidant activity intra- or extracellularly by sequestering/blocking CPZ or oxidative stress-induced molecules, we subjected SH-SY5Y to different pharmacological inhibitors of endocytosis. First, we examined the above-described neuroprotective effect of Apo D against CPZ upon perturbation of clathrin-mediated endocytosis (CME), or upon alteration of actin-dependent phagocytosis and micropinocytosis by pretreatment of cells with chlorpromazine (5 µg/mL) and cytochalasin D (8 µg/mL), respectively. As shown in Figure 10, these conditions did not seem to influence the effect exerted by hApo D (50–100 nM), even they significantly enhance it, as demonstrated by the MTT assay. However, when cells were pretreated with dynasore (80 µM), a compound that blocks GTPase activity of dynamin and vesicle scission, hApo D was not able to prevent the significant decrease of about 20–25% in cell viability after 24 h of treatment with 500 µM CPZ (Figure 10). Nevertheless, it should be noted that dynasore drastically magnifies, in some way, the cytotoxic effect of CPZ.

### 2.5. Neuroprotective Effect of hApo D is not Related to a Decrease in CPZ-Induced ROS Levels

**Figure 11:**
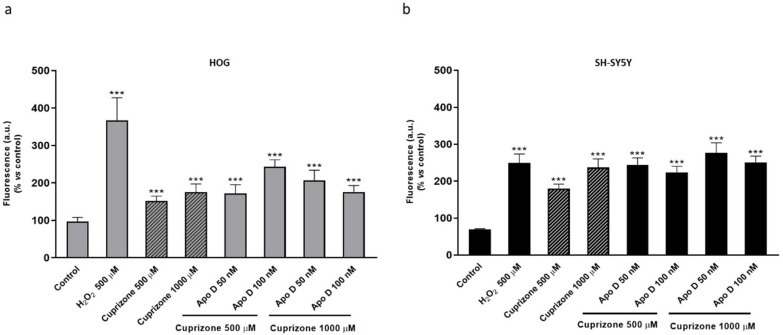
ROS production in HOG (**a**) and SH-SY5Y cells (**b**) treated with increasing concentrations (50–100 nM) of hApo D for 24 h followed by 24 h with 500 or 1000 µM of CPZ. Changes in ROS levels, measured with the oxidant-sensitive dye H_2_DCFDA, are represented as the percentage of fluorescent DCF production versus control. H_2_O_2_ 500 µM was used as positive control. Data are the mean ± SEM of five independent experiments. Significant differences were analyzed by a one-way ANOVA followed by post-hoc Tukey’s test. *** *p* < 0.001 compared with control.

We previously demonstrated that CPZ affects mitochondrial function and aerobic cell respiration in neurons and glial cells which could lead to an increase in intracellular ROS production. In fact, the data summarized in Figure 11 show that treatment of HOG and SH-SY5Y cells with CPZ concentrations of 500 and 1000 µM significantly increased the levels of intracellular ROS. In the MTT assays Apo D totally prevented the loss of viability caused by CPZ so the next step was to measure ROS formation in these conditions. However, we did not find significant differences in the levels of intracellular ROS production between cells pretreated or not with hApo D (Figure 11a,b).

## 3. Discussion

Thirty years of research have provided significant insights to unravel the function of Apo D, which has helped to elucidate its antioxidant and anti-inflammatory role and a better understanding of the mechanisms whereby this specific apolipoprotein may exert its beneficial effects. To elucidate the function of Apo D requires the development of cellular models that allow studying the actions of this protein in a physiologically relevant but simple context. The data here presented aims to make progress in the knowledge of potential neuroprotective effect of Apo D in MS and other demyelinated diseases by both indirect and direct in vitro approximations.

A common trend in multiple pathological and nonpathological conditions of the nervous system, from neural development and aging to diverse neurodegenerative processes such as those observed in MS, is the Apo D upregulation with a seemingly neuroprotective purpose [12,20,51,55,56]. Valuable information has been gained concerning the Apo D expression in MS, i.e., it is increased in the CSF of MS patients and exhibits a characteristic pattern in the brain lesions [49,50,51]. However, mechanisms involved in the Apo D function in this pathology have been not fully elucidated until now. By taking advantage of the CPZ-induced model of MS, we aimed to analyze the expression of Apo D in HOG cells treated with CPZ. Our results showed that the changes induced by CPZ in Apo D expression are minimal despite the dose and time-dependent cytotoxic damage previously reported for CPZ in the same conditions. Unsurprisingly, a similar CPZ effect was demonstrated in the SH-SY5Y, a cell line that does not efficiently express Apo D at least in nonpathological conditions. In fact, we only found negligible levels of this apolipoprotein in the neuroblastoma cells by immunocytochemistry but not by other techniques, probably due to methodological differences. During the last decades, various authors, including us, have used in vitro assays to demonstrate that H_2_O_2_, amyloid beta-peptide, lipopolysaccharide, paraquat as well as other acute short-term oxidative stressors induce a time- and dose-dependent effect on Apo D expression [55,56,57,58,59]. At least in astrocytes, this effect seems to be regulated by the stress responsive JNK signaling pathway [9]. Here, we found that CPZ by itself does not promote a significant increase in Apo D levels in oligodendroglioma cells. Although the exact mechanism of action of CPZ is not completely understood, we previously demonstrated that the ion chelator impacts on functional state of mitochondria and aerobic cell respiration in neurons and glial cells. Now, we have also shown that these processes are accompanied by a significant increase in intracellular levels of ROS. Interestingly, the consequent compromise of mitochondrial function, cell metabolism, and the increase in oxidative stress are not immediately apparent in in vivo CPZ models. In this regard, the toxic/demyelinating effect induced by CPZ in mice does not peak until the third week of treatment [59,60], so it is reasonable to assume that CPZ may trigger the Apo D expression in the longer term.

Neuroprotection by Apo D may be afforded by either an indirect or a direct manner. On the one hand, it has been shown that CLO, an atypical antipsychotic drug used in the treatment of schizophrenia and bipolar syndrome, is able to increase Apo D levels in the brain [53,54]. However, the exact mechanism by which CLO regulates Apo D expression is still unknown. Our findings demonstrated that CLO induces an increase in Apo D synthesis in OLGs and neurons only in the presence of CPZ, at the same time that moderately prevents the loss of viability caused by the toxin, i.e., at neuroprotective doses of CLO. An important aspect, according to the findings obtained in SH-SY5Y cells, is that low concentrations of CLO would be the ones that may exert an Apo D-related neuroprotection against CPZ. In this way, we hypothesize that the great increase in Apo D synthesis induced by the treatment with 5 µM of CLO, unable to prevent the cytotoxic effect of CPZ, may be a consequence of some cell stress/damage caused by this dose of antipsychotic drug.

Based on our data, it seems that Apo D may contribute to the protective effect of the drug. This idea is sustained by some in vivo studies postulating that the effect of CLO in pathological situations would be related to the protective function of Apo D, thanks to its ability to (i) bind hydrophobic ligands, (ii) minimize their release, (iii) prevent their peroxidative degradation, and (iv) stabilize plasma membranes [53,61]. In particular, some authors propose that mechanism of action of CLO depends on the role of Apo D in arachidonic acid metabolism [6,62]. Therefore, targeting neural cells to increase Apo D and prevent further death would be one promising choice for MS modifying approaches.

On the other hand, this study was designed to test the effect of Apo D when it is added exogenously. The results obtained, using either a purified or a recombinant version of the hApo D, were interesting as these compounds afforded some neuroprotection against the CPZ insult. In fact, our analysis revealed that the exogenous addition of hApo D, purified from BCF or produced in a mammalian expression system, induces an increase in cell viability/proliferation in normal conditions. At the same time, this apolipoprotein also completely prevents the mitochondrial damage and loss of viability caused by the treatment with moderate to high doses of CPZ in oligodendroglial cells and, more importantly, in a neuroblastoma cell line that lacks endogenous Apo D expression [52]. Moreover, and in order to check the possibility that Apo D exerts, in this case, its protective activity in an extracellular way, a chemical perturbation of endocytosis in SH-SY5Y cells was carried out. Our data showed that Apo D neuroprotection is largely independent of CME, phagocytosis and macropinocytosis, but it is significantly reduced by inhibitors of dynamin, i.e., dynamin-dependent mechanisms that are consistent with clathrin-independent endocytosis (CIE) modes (caveolae- and RhoA-dependent) [63]. The above-mentioned results may have several implications. First, these results suggest that OLGs and neurons would be able to capture and internalize hApo D from the medium, triggering an increase in the cell metabolic activity and/or proliferation rate. Apo D uptake by some cells is not an unknown phenomenon. For instance, it is clearly demonstrated that astrocytes and OLGs synthesize and secrete this protein [16,18,64] which is captured by certain neurons in some specific situations [9,64,65,66]. Although technically challenging, pioneering studies in last years stated that Apo D may enter cells as a clathrin-independent cargo mediated by a specific cell surface receptor, basignin [67]. The recent discovery that Apo D is located inside the endosome-lysosome-autophagosomal compartment [66] and the results here presented support this hypothesis. Second, the effect of exogenous Apo D, once internalized, turns neuroprotective in pathological situations, which is consistent across studies. For example, Najyb et al. (2017) demonstrated that hApo D internalization and accumulation in primary hippocampal neurons were accentuated by kainate treatment. In addition, these authors reported that hApo D could act by decreasing abnormally increased cholesterol levels in damaged neurons [68]. In this line, He et al. (2009) showed that hApo D purified from BCF was able to bind arachidonic acid and cholesterol, attenuating the increase in oxidants and proinflammatory derivatives as F(2)-isoprostanes and 7-ketocholesterol in similar pathological conditions [69]. These neuroprotective and antioxidant roles of Apo D may be closely associated with its capacity of reducing radical-propagating lipid hydroperoxides by three methionine (Met) residues (Met49, Met93, and Met157), highly conserved in mammals [70]. Alternatively, Apo D has an extra cysteine, Cys116, with a thiol group that can be implicated in a direct antioxidant activity [70,71]. Despite this, and according with our findings, the protective mechanism of Apo D against oxidative damage induced by CPZ may directly target mitochondrial function but would not act through the production levels of ROS.

Noteworthy, although Apo D has been generally described as a monomeric protein [4,72], it dimerizes when reducing peroxidized lipids [7,15]. Thus, small-angle X-ray scattering analysis revealed that this apolipoprotein is mainly present as a tetramer in BCF or an oligomer in CSF [15]. As a general rule, heteromers are currently considered as novel molecular entities with new ligand and signaling characteristics, and probably different antioxidant properties which could explain the greater neuroprotective effect of hApo D, compared with hrApo D, demonstrated here.

In summary, valuable information has been gained in this work concerning neuroprotective effect of Apo D against CPZ, a neurotoxin used to produce models of MS. Although the development of simpler models, as the ones shown in this work, constitutes a way to provide reliable answers in some pathological situations, these results must be validated on more physiological models such as primary cultures in order to check whether increasing either endogenously and/or exogenously the levels of Apo D could be a feasible intervention as part of medical therapy in neurodegenerative diseases. In this regard, the origin and the native structure of this protein must be taken into account in order to design the most effective approach.

## 4. Materials and Methods

### 4.1. Cell Lines

HOG cell line, established from a surgically removed human oligodendroglioma by Dr. A. T. Campagnoni (University of California, UCLA, Berkeley, CA, USA) [73] was kindly provided by Dr. J. A. López-Guerrero (Universidad Autónoma de Madrid, Madrid, Spain) [74]. Cells were grown in DMEM, low glucose, pyruvate, HEPES (22320-022, Invitrogen, Paisley, Scotland, UK), 100 units/mL penicillin/streptomycin (17-602E, Invitrogen, Paisley, Scotland, UK), and 10% (*v*/*v*) heat inactivated fetal bovine serum (FBS) (10270-106, Invitrogen, Paisley, Scotland, UK).

Human neuroblastoma SH-SY5Y cell line was obtained from Sigma (ref 94030304, Sigma-Aldrich, St. Louis, MO, USA) and was grown in DMEM supplemented with 2 mM L-glutamine (61965-059, Invitrogen, Paisley, Scotland, UK), 100 units/mL penicillin/streptomycin (17-602E, Invitrogen, Paisley, Scotland, UK), 1% nonessential amino acids (11140-035, Invitrogen, Paisley, Scotland, UK), and 10% (*v*/*v*) heat inactivated FBS (10270-106, Invitrogen, Paisley, Scotland, UK).

Cells were maintained at 37 °C in a humidified atmosphere of 5% CO_2_ and were passaged when they were 80–90% confluent, i.e., approximately twice a week for no more than 20 passages.

### 4.2. Human Apo D Purification

Human Apo D (hApo D) was purified from BCF samples provide by the Pathology Unit of the Hospital Universitario Central de Asturias (HUCA). First, a cell fractionation was performed by differential centrifugation. Then, Amicon^®^ Ultra-15, 100 kDa, centrifugal filter units (Z740211, Sigma-Aldrich, St. Louis, MO, USA) were used. The solution containing the protein was flow through two consecutively ion-exchange chromatographic columns (HiTrap^®^ Q Fast Flow, GE Healthcare, Chicago, IL, USA) with 25 mM Tris pH 8.0, followed by a size-exclusion chromatography (HiLoad^®^ 16/60 Superdex^®^ 200 prep grade, GE Healthcare, Chicago, IL, USA ) in 50 mM Tris pH 8.0, 75 mM NaCl. Elution fractions with the protein of interest can be further concentrated using an appropriate 30 kDa cut-off Amicon^®^ centrifuge filter (Z717185, Sigma-Aldrich, St. Louis, MO, USA). The presence of hApo D in these fractions was checked by Western blot, the amount of this apolipoprotein (concentration in the fraction) was quantified and its functionality was tested.

### 4.3. Cell Treatments

**Figure A3:**
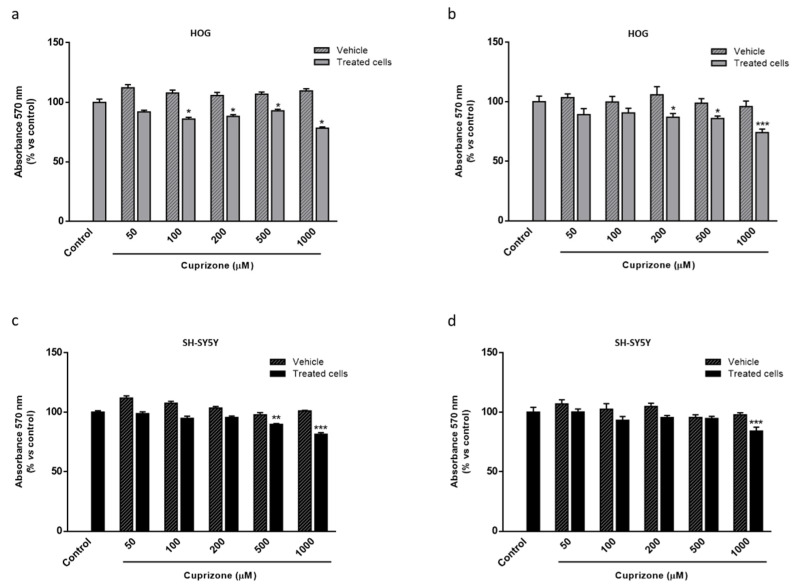
MTT assay in HOG (**a**,**b**) and SH-SY5Y (**c**,**d**) cells treated with increasing concentrations of CPZ (50–1000 µM) and their respective vehicles for 24 h (**a**,**c**) or 48 h (**b**,**d**). Cell damage is represented as the percentage of viability versus control. Data are the mean ± S.E.M of five independent experiments. Significant differences were analyzed by a one-way ANOVA followed by post-hoc Tukey’s test. * *p* < 0.05, ** *p* < 0.01, *** *p* < 0.001 compared to control.

For CPZ treatment, a stock solution of 30 mM CPZ (C9012-25G, Sigma-Aldrich, St. Louis, MO, USA) was prepared freshly. For this, CPZ powder was dissolved in 50% ethanol/medium and shaken at 225 rpm at 60 °C for 15–20 min until its complete dissolution. Working solutions were prepared by diluting the stock in the specific medium for each cell type in a series of sequential solutions to reduce the ethanol concentration [72,73]. After 24–48 h of plating (30−40% cellular confluence), cellular toxicity was induced by the addition of CPZ in growing concentrations (50–1000 µM; see corresponding figure legends), for 24, 48, or 72 h. In order to prove that results are only attributable to CPZ not ethanol, the vehicle effect was also tested for each sequential solution in the cell models. As it can be observed in the graphs (Figure A3), we demonstrated that even the highest concentration of ethanol used to dissolve CPZ did not negatively affect, in a statistically significant way, cell viability of HOG and SH-SY5Y cells after 24 and 48 h of treatment.

To investigate the effect of the antipsychotic drug CLO (C6305-100G, Sigma-Aldrich, St. Louis, MO, USA) in the Apo D expression, cells were treated with different concentrations (0.1–5 nM; see corresponding figure legends) for 24 h, before fresh addition of CPZ. For the exogenous addition of Apo D, hApo D purified from BCF or hrApo D derived from human cells (P05090, Novoprotein, Summit, NJ, USA) were added (0.05–1000 nM; see corresponding figure legends) to cell cultures 24 h prior to CPZ.

For inhibition of endocytic mechanisms, SH-SY5Y cells were treated with different chemical inhibitors, cytochalasin D (8 µg/mL; C2618, Sigma-Aldrich, St. Louis, MO, USA), chlorpromazine hydrochloride (5 µg/mL; C8138, Sigma-Aldrich, St. Louis, MO, USA) and dynasore (80 µM; 324410, Sigma-Aldrich, St. Louis, MO, USA). Stock solutions were prepared in dimethyl sulfoxide (DMSO) and diluted in serum-free medium supplemented with 30 mM HEPES on the day of the experiment. The final DMSO concentration added was kept <0.1%. Cells were washed in serum-free medium and treated with the respective inhibitors (see corresponding figure legends) for 30 min before Apo D addition. H_2_O_2_ was used as positive control.

Drug concentrations and times of treatments were based on the bibliography and on our previous experience [75,76].

### 4.4. MTT Assay

Cell viability was studied by 3-(4,5-dimethylthiazol-2-yl)-2,5-diphenyltetrazolium bromide (MTT) reduction assay, a method based on the activity of mitochondrial NAD dependent oxidoreductases as indicator of the functional state of mitochondria. For this, 3000–5000 cells/well were seeded in 96-well plates and grown in 100µL/well of complete medium. Once treatments were completed, 10 μL of MTT (5mg/mL in phosphate buffered saline (PBS; 10010-023, Gibco, Invitrogen, Paisley, Scotland, UK)) (M5655; Sigma-Aldrich, St. Louis, MO, USA) were added to each well. Four hours later, 100 μL of lysis solution (20% sodium dodecyl sulfate (SDS); 50% dimethylformamide; pH 4) were added to the culture and incubated overnight at 37 °C. Absorbance at 570 nm was measured using a Multiskan EX Microplate Reader (ThermoFisher Scientific, Waltham, MA, USA). Values from blank wells, containing only medium, were subtracted from the values of the samples. Cell viability was expressed as the percentage of the controls.

### 4.5. Determination of ROS

The intracellular level of ROS, as an important biomarker for oxidative stress, was estimated with the dye 2′7′-dichlorodihydrofluorescein diacetate (H_2_DCFDA; D399, Molecular Probes, Invitrogen, Paisley, Scotland, UK), a nonpolar compound that easily penetrates into the cell where it is hydrolyzed to the nonpermeant H_2_DCF. This nonfluorescent compound becomes oxidized by various ROS to highly fluorescent 2′,7′-dichlorofluorescein (DCF). For determination, 3000–5000 cells/well were seeded in 96-well plates and grown in 100 µL/well of complete medium. Once treatments were completed, medium was removed and prewarmed PBS containing the probe (final working concentration of 10 µM dye) was added to the cells. After incubation for 60 min at 37 °C, the dye was removed and cells were returned to prewarmed growth medium. Then, fluorescence was measured in a microplate fluorimeter FLX-800 (Bio-Tek Instruments, Inc., Winooski, VT, USA) at an excitation wavelength of 485 nm and an emission wavelength of 528 nm.

### 4.6. Immunocytochemistry

Cells were seeded over glass coverslips (10 mm diameter) in 6-well plates at a density of 50,000 cells/well in a final volume of 2 mL of medium. Once the treatments were concluded, cells were washed three times with PBS and fixed in bouin solution for 15 min. After fixation, cells were washed three times and then permeabilized by incubation with 1% Triton X-100 at room temperature for 15 min. Nonspecific binding was blocked by incubation with bovine serum 30 min at room temperature. Incubation with anti-human Apo D antibody 1:2000 (provided by Dr. Carlos López-Otín, department of Biochemistry and Molecular Biology, University of Oviedo; see [58,77,78]) was carried out overnight in a humid chamber at 4 °C. After three washes in PBS, coverslips were incubated 30 min at room temperature using a biotinylated horse universal antibody (Universal quick, PK-8800, Vector Laboratories, Inc., Burlingame, CA, USA) diluted 1:50. After that, cells were incubated with streptavidin Alexa Fluor^®^ 550 conjugate (1:500; S2138, Invitrogen, Paisley, Scotland, UK). Finally, cells were washed in distilled water, dehydrated, cleared in eucalyptol and mounted with Fluoromount. The fluorescence was visualized in a Nikon Eclipse E400 microscope equipped with a Nikon G2-A and recorded by a digital camera (Nikon DN-100). The resulting immunocytochemical signal was selected with Photoshop and quantified with ImageJ 1.57 software (NIH, Bethesda, MD, USA) [79]. Images were acquired under the same conditions of illumination, diaphragm and condenser adjustments, exposure time, and background correction. For control purposes, representative cell cultures were processed in the same way with a nonimmune serum or with specifically absorbed sera instead of the primary antibody. Under these conditions no specific immunostaining was observed.

### 4.7. RNA Purification

Cells were recovered from culture dishes using scrapers and TRIzol (15596018, Invitrogen, Paisley, Scotland, UK), then total RNA was purified using the RNeasy mini-kit (74104, Qiagen, Valencia, CA, USA) with a DNAse digestion step performed (79204, Qiagen, Valencia, CA, USA) following the manufacturer’s instructions.

### 4.8. Quantitative Real-Time PCR

Random primers and the SuperScript III kit (11752050, Invitrogen, Paisley, Scotland, UK) were used to reverse-transcribe 1 µg of total RNA into first-strand cDNA in a total volume of 20 μL according to the manufacturer’s instructions. SYBR Green PCR Master Mix (a25778, Applied Biosystems, Carlsbad, CA, USA) was mixed with cDNA for quantitative real time polymerase chain reaction (qRT-PCR) using 0.3 µM forward and reverse oligonucleotide primers (Table 1). 7300 Real Time PCR System (Applied Biosystems, Carlsbad, CA, USA) was used for quantitative measure of gene expression. Cycling conditions were an initial denaturation at 95 °C for 10 min, followed by 40 cycles of 95 °C for 15 s 60 °C for 1 min. At the end, a dissociation curve was implemented from 60 to 95 °C to validate amplicon specificity. Relative quantification of gene expression was calculated by interpolation into a standard curve. All values were divided by the expression of the house keeping gene 18S rRNA.

### 4.9. Data Analysis

The data in the graphs are presented as the mean ± S.E.M, from at least five independent experiments. The normality of population and the homogeneity of variance were evaluated by the test of Kolmogorov–Smirnov with the correction of Lilliefors and the test of Levene, respectively. Then one- or two-way ANOVA tests followed by post hoc Tukey’s test for multiple comparisons were used to compare the values. Statistical analysis was carried out with SPSS 18.0 software (IBM, Armonk, NY, USA). Significant differences were considered when *p* < 0.05.

## Figures and Tables

**Table 1:** Primers used for qRT-PCR in this study.

Oligonucleotide		Sequence
R2-ApoD-F	--------	TGCATCCAGGCCAACTACTC
R2-ApoD-Rev	--------	GGGTGGCTTCACCTTCGATT
18S-Fw	--------	ATGCTCTTAGCTGAGTGTCCCG
18S-Rev	--------	ATTCCTAGCTGCGGTATCCAGG

The annealing temperature was 60 °C for all primers. 18S rRNA was used as a housekeeping gene.

## Data Availability

The data presented in this study are available on request from the corresponding author. The data are not publicly available due to privacy restrictions.

## Abbreviations

AD	Alzheimer’s disease
Apo D	Apolipoprotein D
BBB	Blood–brain barrier
BCF	Breast cyst fluid
CIE	Clathrin-independent endocytosis
CLO	Clozapine
CME	Clathrin-mediated endocytosis
CNS	Central nervous system
CPZ	Cuprizone
CSF	Cerebrospinal fluid
DCF	2′,7′-dichlorofluorescein
DMSO	Dimethyl sulfoxide
FBS	Fetal bovine serum
H_2_DCFDA	2′7′-dichlorodihydrofluorescein diacetate
hApo D	Human Apo D
HDL	High-density plasma lipoproteins
hrApo D	Human recombinant Apo D
HUCA	Hospital Universitario Central de Asturias
MS	Multiple sclerosis
MTT	3-(4,5-dimethylthiazol-2-yl)-2,5-diphenyltetrazolium bromide
OLGs	Oligodendrocytes
PBS	Phosphate buffered saline
PD	Parkinson’s disease
PNS	Peripheral nervous system
RNS	Reactive nitrogen species
ROS	Reactive oxygen species
SDS	Sodium dodecyl sulfate
UV	Ultraviolet
